# Malaria Diagnosis across the International Centers of Excellence for Malaria Research: Platforms, Performance, and Standardization

**DOI:** 10.4269/ajtmh.15-0004

**Published:** 2015-09-02

**Authors:** Tamaki Kobayashi, Dionicia Gamboa, Daouda Ndiaye, Liwang Cui, Patrick L. Sutton, Joseph M. Vinetz

**Affiliations:** Department of Epidemiology, Johns Hopkins Bloomberg School of Public Health, Baltimore, Maryland; Laboratorio de Malaria, Laboratorios de Investigación y Desarrollo, Facultad de Ciencias y Filosofía, Universidad Peruana Cayetano Heredia, Lima, Peru; Institute de Medicina Tropical, Universidad Peruana Cayetano Heredia, Lima, Peru; Department of Parasitology and Mycology, Faculty of Medicine, Pharmacy, and Odontology, Université Cheikh Anta Diop de Dakar, Dakar, Senegal; Department of Entomology, Pennsylvania State University, University Park, Pennsylvania; Center for Genomics and Systems Biology, New York University, New York, New York; Department of Biology, New York University, New York, New York; Division of Infectious Diseases, Department of Medicine, University of California San Diego School of Medicine, La Jolla, California

## Abstract

Diagnosis is “the act of identifying a disease, illness, or problem by examining someone or something.” When an individual with acute fever presents for clinical attention, accurate diagnosis leading to specific, prompt treatment often saves lives. As applied to malaria, not only individual patient diagnosis is important but also assessing population-level malaria prevalence using appropriate diagnostic methods is essential for public health purposes. Similarly, identifying (diagnosing) fake antimalarial medications prevents the use of counterfeit drugs that can have disastrous effects. Therefore, accurate diagnosis in broad areas related to malaria is fundamental to improving health-care delivery, informing funding agencies of current malaria situations, and aiding in the prioritization of regional and national control efforts. The International Centers of Excellence for Malaria Research (ICEMR), supported by the U.S. National Institute of Allergy and Infectious Diseases, has collaborated on global efforts to improve malaria diagnostics by working to harmonize and systematize procedures across different regions where endemicity and financial resources vary. In this article, the different diagnostic methods used across each ICEMR are reviewed and challenges are discussed.

## Introduction

Combating the global malaria burden begins with accurate diagnosis, which guides specific treatment and public health reporting. Reliable malaria diagnosis improves health-care delivery and informs funding agencies of current malaria situations, which is key for prioritization of regional- and national-level control efforts. Adequate malaria diagnostics contribute toward the long-term goal of malaria elimination, but accuracy in both testing and reporting depends on resource availability, health worker expertise, and formal reporting policies that vary among regions. Microscopy and rapid diagnostic tests (RDTs), which detect parasite antigen, are most commonly used to diagnose malaria; each method has its own strengths and weaknesses.^1^ Current World health Organization (WHO) recommendations for malaria diagnosis focus on the identification of acute symptomatic malaria.^2^ Therefore, individuals with subclinical malaria parasitemia who do not present at health facilities, and those with parasitemia below the detection limit of microcopy or RDTs, are missed.^3^,^4^ Individuals with sub-patent, subclinical parasitemia are increasingly recognized as key for maintaining regional malaria transmission, and as malaria transmission declines in some areas because of increased malaria control, such individual contributions to malaria endemicity become increasingly important.^3^,^5^,^6^ Because regional malaria transmission profiles will change as control strategies progress to elimination, diagnosing these changes will become necessary on a global level. Consequently, the National Institute of Allergy and Infectious Diseases established the International Centers of Excellence for Malaria Research (ICEMR) program in 2010. As summarized in the foreword of this journal supplement, the ICEMR network includes 10 independent research programs representing all major malaria-endemic regions. Integral to each ICEMR is the inclusion of multiple, epidemiologically contrasting field sites and innovative multidisciplinary approaches and the use of robust diagnostic strategies to help assess the changing malaria situation in each endemic region. The goal of this integrated approach is to generate a knowledge base for improving clinical and field management of malaria. In this article, we discuss laboratory-based diagnostic tools used by ICEMRs, which is a major focus of their research programs.

Since 2010, WHO has recommended either RDT or microscopy confirmation of suspected malaria cases before treatment.^2^ The use of RDTs increased globally from less than 200,000 in 2005 to more than 74 million in 2011.^7^ The increased availability and use of RDTs in the public sector has resulted in fewer cases of non-malaria acute febrile illness being empirically treated with antimalarial drugs, which helps to prevent development of drug resistance. Although both microscopy and RDTs are of great value in guiding appropriate malaria treatment, subclinical/sub-patent *Plasmodium* parasitemic individuals do not come to clinical attention and cannot be detected by these techniques because of limited sensitivity.^3^,^5^,^6^

During the past decade, highly sensitive and specific nucleic acid amplification techniques have been developed to detect malaria parasites including polymerase chain reaction (PCR), real-time quantitative PCR (qPCR), and reverse transcriptase PCR (RT-PCR) (reviewed in references^1^,^8^). Compared with light microscopy's limit of detection (about 30 parasites/μL by the best microscopists) or RDTs (> 100 parasites/μL), nucleic acid amplification methods can detect fewer than 10 parasites/μL.^1^,^8^ PCR-based methods are extensively used by ICEMRs for the field of malaria epidemiology research. Highly sensitive qPCR methods were developed by either increasing the volume of sample extracted and analyzed^9^ or by targeting multi-copy genes.^10^ However, such techniques often require sophisticated equipment and training and are significantly more expensive than microscopy and RDT. To make highly sensitive but technologically intense malaria diagnostics more readily available in the resource-limited setting, isothermal amplification techniques were adapted.^11^ Loop-mediated isothermal amplification (LAMP), the best characterized of these techniques, is as sensitive and specific as conventional PCR without needing sophisticated equipment for extraction, amplification, or detection.^12^,^13^ Comparison of the detection limit between various PCR methods and isothermal detection methods has shown that the lower detection limit of LAMP is 5–10 parasites/μL, comparable to that of conventional PCR.^11^ Other isothermal techniques such as nucleic acid sequence–based amplification (NASBA) can achieve detection limit of < 1 parasite/μL on blood sample sizes of at least 50–100 μL.^14^,^15^ Other molecular approaches can be used to detect malaria parasites (Table 1). For field-based epidemiology studies, a major focus area of ICEMR efforts, PCR-based methods are most commonly used to detect, quantify, and speciate low-density malaria parasitemia, whether asexual or gametocyte forms. Because of space limitations here, we primarily focused on PCR-based methods currently used by the ICEMR network.

Malaria control and elimination strategies depend on providing appropriate treatment of parasitologically confirmed clinical malaria cases as well as asymptomatic carriers. Such specific treatment depends not only on accurate diagnostics but also on effective (and pure or “valid”^16^) drugs.^17^ Throughout the malaria-endemic world, *Plasmodium falciparum* has developed some level of resistance to many current antimalarial drugs, and chloroquine-resistant *Plasmodium vivax* has been reported in some regions.^18^ To address multidrug resistance malaria parasites, WHO has recommended the use of artemisinin-based combination therapy (ACT) to treat *P. falciparum* in most malaria-endemic regions.^19^ In areas of *P. vivax* chloroquine resistance, ACTs are also recommended or deployed for treatment of vivax malaria.^20^,^21^ Because of the high demand and its market value, there has been an issue with counterfeit ACTs.^22^,^23^ A field deployable method to test drug potency is needed to help prevent the emergence of parasite drug resistance. Recent work by the southeast Asia ICEMR toward this end, to develop an assay to verify the quality of artemisinin class antimalarials, is discussed later in the section, RDT to check quality of artemisinin class antimalarials.

## The Diagnostic Dilemma and Currently Available Platforms

Accurate diagnosis is essential for treating suspected malaria cases, but in many malaria-endemic regions fever is commonly presumed to be malaria without confirmation.^24^ A diagnostic test for malaria must be specific to identify the infecting *Plasmodium* species, readily available with a rapid turnaround time, and inexpensive. Even though malaria testing in Africa focuses on *P. falciparum*, effective diagnostics for *P. vivax* are important elsewhere because parasitemia is typically lower. Identifying very low parasitemia individuals is not feasible with RDTs, but requires highly competent microscopy complemented by molecular assays. Currently, it is not possible to diagnose asymptomatic *P. vivax* (and *Plasmodium ovale*) hypnozoite carriers, yet developing methods to do so is important because the biology of *P. vivax* makes elimination of this parasite more difficult.^18^,^25^–^27^ Even though diagnostic strategies in some countries have started to use combination RDTs more often, RDTs are not useful in elimination settings, but only for diagnosis of acute disease. Further such strategies do not take the diagnosis of *P. ovale* or *Plasmodium malariae* into account, particularly in Africa, or the zoonotic *Plasmodium knowlesi* in Asia. It is important to identify infections due to both single and more than one *Plasmodium* species to ensure proper antimalarial drug treatment.^2^ *P. vivax* and *P. ovale* form dormant hypnozoites that should be treated with an 8-aminoquinoline (primaquine; tafenoquine in clinical trials), despite challenges related to using these medications on population scales because of the potential for adverse events due to the presence of even small percentages of people having some form of glucose-6-phosphate dehydrogenase deficiency.^18^,^28^–^33^ On a population and geographic level, antimalarial treatment carried out without regard to specificity of diagnosis is a major risk for drug tolerance and resistance as well as potential clinical complications, all of which are concerns throughout the malaria-endemic world. With regard to population-level reservoirs of continuing transmission, a specific diagnostic test must be able to identify sub-patent cases, defined as infections with parasite levels below the limit of microscopic or RDT detection.^5^ Sub-patent parasitemia cases likely contribute to transmission in endemic regions.^3^,^6^ Furthermore, sub-patent parasitemia cases are often subclinical, and inaccurate detection of subclinical cases, either due to insufficiently effective diagnostics or policies that impede the diagnosis and treatment of subclinical cases, interferes with malaria control efforts in regions, particularly those nearing elimination. The superior sensitivity of molecular diagnostics could ameliorate both of these problems, and provides opportunity for researchers to investigate other important epidemiological questions, including exposure frequency and the transmissibility of malaria parasites based on the quantification of sexual stages.

Currently, there are many molecular diagnostics platforms being used in the ICEMR network (Table 1). Even though their performance has many advantages, most molecular diagnostics are either unable to be sustained in the field because of cost/resource availability or too laborious to be effective for point-of-care diagnosis, hence reducing effectiveness. RDTs have been useful for point-of-care diagnosis, but the issue of false positive results due to the residual antigenemia after the clearance of the parasite remains problematic.^34^–^36^ Because of low sensitivity, RDTs are not suitable for diagnosis of very low parasitemias. The limitations of molecular diagnostics and RDTs make the continued use of light microscopy important for accurate diagnosis.

## Cross-ICEMR Comparison

### Diagnostic methods.

The ICEMR program was designed to include a wide range of malaria transmission intensities in contrasting epidemiological settings. Studies were required to be designed to identify and quantify the *Plasmodium* species causing malaria toward a more complete understanding of the complex interactions among human hosts, malaria parasites, and mosquito vectors in diverse ecological niches taking into account expected and unexpected changes occur over space and time. All 10 ICEMRs implemented light microscopy–based diagnosis as the primary quantification metric. Nine study sites used microscopy plus RDTs; Amazonia ICEMR did not use RDTs for several reasons: microscopy is generally available and accurate; the dominance of *P. vivax* compared with *P. falciparum* malaria, combined with the lactate dehydrogenase (LDH)–based RDTs that were not considered sufficiently sensitive; and national policy requires microscopy-based diagnosis. In Peru, one country in which the Amazonia ICEMR is based, *pfhrp2*- and/or *pfhrp3*-lacking *P. falciparum* were first reported, which made the histidine-rich protein 2/3 (HRP2/3)–based RDT inadequate for use as described later in the section, HRP2/3 deletion among the circulating parasites. Among ICEMRs, the choice of different RDTs has been largely dependent on availability and national policy–based recommendations (Table 2). ICEMRs reporting the use of RDT were aware of their limitation. There, RDTs are meant to be used for clinically apparent cases, primarily where *P. falciparum* is the major parasite and where parasitemia is sufficiently high to enable RDT detection. The sensitivity of RDTs is not sufficient for screening of subclinical cases where the parasitemia is generally very low, a focus of most ICEMRs. All 10 ICEMRs implemented molecular diagnostic methods either by conventional PCR, qPCR, or RT-PCR (Table 2). In addition, half of the ICEMRs have also implemented gametocyte detection by sexual stage (*Pfs25*, *Pvs25*)–specific RT-PCR (Table 2). Two of the 10 ICEMRs use nucleic acid amplification techniques as a point-of-care diagnosis (i.e., southern Asia ICEMR and west Africa ICEMR), whereas the majority of the ICEMRs use nucleic acid amplification techniques only in the research setting (Table 2). The detection limit reported by the majority of ICEMRs was 11–50, 51–200, and < 10 parasites/μL for microscopy, RDTs, and molecular diagnostics (i.e., LAMP, conventional PCR, qPCR, and gametocyte-specific RT-PCR), respectively.

For the detection of malaria parasite DNA, the majority of ICEMRs reported the use of the 18S rRNA as a target gene either by conventional PCR or qPCR to identify malaria species (Table 2).^37^–^39^ Other target genes such as *pfr364*, *cytb*, and *pfldh* were also used, but less frequently (Table 2).^40^,^41^

### Factors affecting the sensitivity of different diagnostics.

Microscopy and RDTs are extensively used to diagnose malaria at health facilities. Because of extensive experience, quality assurance/quality control (QA/QC) protocols are well established to assure reliable and comparable results. For light microscopy, double reading of the slides with a third reader for discrepancies is standard among ICEMRs. WHO recommends that parasite quantification be performed against 200 white blood cells (WBCs), and if the parasite count is less than 100 after counting 200 WBCs, the microscopist should continue to 500 WBCs.^42^ The west Africa ICEMR reported implementation of minimum count of 300 WBCs per specimen can improve the sensitivity of the slide reading (D. Krogstad, unpublished data). Increasing the WBC count ensures that low-parasitemia cases are not missed, but requires a substantial increase in microscopy time.

WHO collaborates with the Foundation for Innovative New Diagnostics to evaluate and standardize the use of RDTs.^43^ For molecular diagnostics, standardization has not been established. The decision to choose a protocol and target genes to use largely depends on the research hypothesis. Nine of the ICEMRs use conventional PCR or qPCR to detect, quantify, and speciate submicroscopic parasitemia. RT-PCR coupled with qPCR is used to detect and quantify the gametocyte stage of the parasite with high sensitivity.^5^

### Sample preparation.

All 10 ICEMRs currently use at least one of the molecular diagnostic methods such as conventional PCR, qPCR, and LAMP (Table 2). Suitable sample preparation (i.e., DNA extraction) enables optimal sensitivity and is probably the most important variable to affect field deployability. Various DNA extraction methods, which ultimately depend on starting material, have been reported (Table 3). The methods that were reported to be used by ICEMRs are shown in italics in Table 3. The factors influencing the sensitivity of molecular diagnostics, particularly for conventional PCR, are reviewed in brief, but individual ICEMR protocols are not detailed here.

Dried blood spot (DBS) sampling is commonly used to preserve whole blood samples because it is an easy, durable method with an efficient use of space and optimal transport conditions. In research setting, DBSs have been used as starting material for PCR, RT-PCR, and serology.^59^–^61^ Four ICEMRs have collected DBSs and whole blood as packed red cells, five ICEMRs collected only DBS, and one ICEMR collected only whole blood as packed red cells but not DBSs. DNA extraction differed among ICEMRs. Three of the 10 ICEMRs used a Chelex-based extraction method^49^; the remaining seven ICEMRs used commercial DNA extraction kits, particularly individual spin column–type kits from Qiagen (Hilden, Germany) (Table 3). When DBSs were used as starting material for DNA extraction, the number/volume of blood spots varied between ICEMRs and the reported amount varied between using a partial blood spot to using more than one blood spot. One full blood spot is thought to typically correspond to approximately 50 μL blood. The available sample volume is affected by how blood is collected, whether by finger prick or venipuncture. With venipuncture, a higher volume of blood is typically available, therefore the starting sample volume is more standardized.

### HRP2/3 deletion among the circulating parasites.

HRP2 is a protein found only in *P. falciparum* asexual stages and young gametocyte stages. Hence HRP2 has been used in RDTs to detect *P. falciparum*.^62^ *Plasmodium* LDH (pLDH), from the glycolytic pathway, is found in all malaria species and is the second most commonly used RDT antigen.^62^ A third target antigen for RDTs is aldolase, another glycolytic enzyme.^62^ Several studies have compared RDTs based on these antigens, with different results in terms of sensitivity, specificity, and positive and negative predictive values.^63^,^64^ The majority of RDT experience in the field setting is in the detection of *P. falciparum*.

RDTs are particularly useful where microscopy is not available, whether because of financial constraints or lack of equipment or sufficiently trained personnel. RDT performance in the field is influenced by many factors such as manufacturing quality, handling and storage conditions, user interpretation, parasite density and limits of detection (which partly depend on parasite biology, e.g., *P. vivax* parasitemia is characteristically low), and the recent discovery of some region-specific field isolates lacking the HRP2 protein. In 2010, *P. falciparum* lacking *pfhrp2* and/or *pfhrp3* genes were first isolated from infected human subjects in the Amazon region of Peru.^65^ Other endemic regions also reported false negative results using RDTs based on PfHRP2 due to *pfhrp2* and/or *pfhrp3* gene deletions.^66^–^68^ The presence of *pfhrp2*/*3* deletion in *P. falciparum* strains has serious implications for diagnosis especially for countries and regions where the antimalarial treatment is primarily based on RDT diagnosis. Therefore, it is important to monitor the prevalence of *pfhrp2* and/or *pfhrp3* gene deletions in regions where RDTs based on PfHRP2 are the primary mode of malaria diagnosis, given the potential need to base procurement decisions on specific RDT detection proteins.

Peru was the first country where *pfhrp2*- and/or *pfhrp3*-lacking *P. falciparum* was identified in infected patients.^65^ These data indicated that the deletion was widespread throughout the Peruvian Amazon region, which had major implications for miscalculated endemicity based solely on RDT detection.^65^ The *pfhrp3* deletion was the most prevalent (70%) compared to *pfhrp2* (41%), and 22% of isolates had both genes deleted.^65^ Peru is not likely the only country affected, as the Amazon River basin runs through other malaria-endemic countries, including Colombia, Ecuador, Brazil, Venezuela, Guyana, Suriname, and Bolivia. Recent reports from these areas showed variable results with regard to the presence/absence of *pfhrp2* and/or *pfhrp3*.^69^–^71^ *Plasmodium falciparum* with the double deletion of *pfhrp2* and *pfhrp3* was found in a patient who returned to Europe from Brazil and misdiagnosed using an RDT based on aldolase and PfHRP2; this patient was treated as having *P. vivax*.^69^ Colombia also reported two *P. falciparum* isolates from clinically infected subjects; however, it was not possible to amplify exon 2 for both *pfhrp2* and *pfhrp3*.^70^ In a recent survey in communities around Iquitos-Nauta road in the Peruvian Amazon region, it was difficult to find *P. falciparum* PfHRP2-positive individuals even though they were diagnosed positive by light microscopy and pLDH (D. Gamboa, A. Llanos, and J. M. Vinetz, unpublished data). Thus far, Guyana is the only country in South America where all *P. falciparum* isolates have been found to carry *pfhrp2*; therefore, to date, the performance of PfHRP2-based RDTs here has not yet been affected.^71^

## RDT to Check Quality of Artemisinin Class Antimalarials

Because they are relatively expensive and widely used, ACTs have become a target of counterfeiters.^22^,^23^ Counterfeit artemisinins have the potential to be a public health and clinical threat because they contain little to no active ingredient hence providing inadequate treatment.^72^ Substandard drugs not only compromise the expected therapeutic effects, but also facilitate the development of resistance.^73^ Although counterfeit artemisinins are known to be a problem in the Greater Mekong subregion of southeast Asia,^74^–^80^ the Worldwide Antimalarial Resistance Network database has reported that poor-quality artemisinins are also a growing concern in Africa.^81^–^85^ Counterfeit and substandard antimalarials are an immediate and urgent threat to the current momentum of global malaria control and elimination efforts. Artemisinin counterfeiting is becoming more sophisticated. Thus far, as many as 14 different formulations of fake artesunate have been identified.^72^,^86^,^87^ To combat the entry of both counterfeit and substandard artemisinin compounds, both national and international regulatory authorities need to strengthen drug quality monitoring to deter the introduction and circulation of poor-quality antimalarials. Until recently, the accurate determination of artemisinin contents in commercial drugs has required sophisticated instrumentation and expertise. The recent development of simple and field-applicable tests to determine the quality of artemisinin drugs is being investigated.

Given that workers in malaria-endemic populations are very familiar with using RDTs for malaria diagnosis, the southeast Asia ICEMR chose to develop a lateral flow dipstick as the point-of-care tests for qualitative and semiquantitative detection of artemisinins in antimalarial drugs. Specific antibodies are needed for such dipstick assays, hence new monoclonal antibodies have been made for such efforts, which include the differentiation of commonly used artemisinin derivatives including artemether, artesunate, and dihydroartemisinin. The first approach was to immunize mice with artesunate conjugated to bovine serum albumin at the succinate group, which yielded a monoclonal antibody (mAb) that reacted with artemisinin, artesunate, and dihydroartemisinin, but with limited reactivity to artemether,^88^,^89^ and a separate mAb that was specific for only artesunate (L. Cui, unpublished data). To achieve specificity for different artemisinin derivatives, which differ in R-groups at position 12, conjugation of the artemisinin derivatives to a carrier protein at the opposite position is needed. For artemether, this is achieved through microbial fermentation of artemether and purification of 9-hydroxylartemether.^90^ A prototype dipstick was designed based on one selected mAb that was found to have high avidity and broad reactivity for artemisinins, with sensitivity as low as 100–200 and 200–500 ng/mL for artesunate and dihydroartemisinin, respectively.^91^ Testing these new tools for monitoring quality of artemisinin drugs is underway. Having specific mAbs in hand for most artemisinin derivatives also allows for enzyme-linked immunosorbent assay quantification.

## QA/QC System for Diagnostic Systems and Implementation in ICEMR

All aspects of malaria control and elimination must have robust quality management and control standards. The concept of quality management for malaria diagnostics covers the entire process including establishment of standard operating procedures for each method and technique used in diagnosis, especially sample tracking and handling, to achieve accurate and reliable test results and reporting. QA aims at improving and standardizing each component of this complicated process to minimize or avoid unreliable results. QC emphasizes test accuracy and precision.^92^ The goal of QC in diagnosis is to detect, identify, evaluate, and correct errors due to technical failure, environmental conditions, and/or human error.

ICEMR sites have established several quality control strategies, most of which aim to comply with WHO guidelines (Table 2).^42^,^93^ In all ICEMR sites, slides are read by two microscopists and a third reader in case of discrepancy (Table 2). Discrepancies in slide reading are usually due to differences in parasite detection limits, species identification, and parasite quantification. In most cases, if there is a disagreement in parasite counts of more than 20% between the two readers, a third reader is consulted to resolve discrepancies.^42^ In some areas, more stringent rules may apply; for example in the Peruvian ICEMR the acceptable discrepancy is ≤ 5%.

To maintain microscopy quality, refresher training programs for microscopists using reference slides as recommended by WHO were reported.^42^,^93^ For any malaria diagnostic laboratory, one way to promote standardization is to request that slide readers pass the WHO accreditation course and to implement regular refresher training and continuing assessments for microscopists. Another QC tactic to help assure microscopy result quality is to have slides reexamined by microscopists at an external reference laboratory or a collaborating institution.

For molecular diagnosis, the inclusion of well-characterized negative and positive controls (including serial dilutions of positive controls for qPCR) was reported from all ICEMRs. Protocols to freshly extract positive and negative controls to assure efficiency and lack of contamination in processed samples remain to be standardized. WHO provides international standards for *P. falciparum* DNA,^94^ and the Malaria Research and Reference Reagent Resource Center also provides useful reagents and other resources. To verify PCR results, some ICEMRs reported sending a fraction of the samples to be reanalyzed at a reference laboratory or a collaborating institution (i.e., Latin America ICEMR and Amazonia ICEMR). Specificity may be increased by performing more than one PCR assay for the identification of sub-patent infection. Although a formal external QA/QC program is not yet available, various attempts to establish such processes have been made, especially for clinical trials and eradication surveillance.^95^,^96^ As qPCR and other more advanced technologies are increasingly used in malaria epidemiology, there is a need to ensure reproducibility of data. A recent article compared different protocols using the WHO International Standard for *P. falciparum* DNA,^97^ leading to a guideline for the minimum information for publication of real-time qPCR experiment. In such a way, the reproducibility of the protocols across different laboratories can be improved.^98^

## Overcoming Key Gaps

Although WHO has published standardized protocols for microscopy and RDTs, no such protocols exist for nucleic acid amplification–based diagnostic tests such as PCR. Each ICEMR site has established conventional PCR; variation in details of sample preparation (starting material volume, nucleic acid extraction methods) remains a challenge for effective comparisons of diagnostic and treatment outcomes data. Other molecular diagnostic tools, such as qPCR, LAMP, and other sensitive techniques, are either not yet available in all the ICEMR regions or were not selected as part of the research design.

International standardization of diagnostic platforms in settings and research networks such as the ICEMR will be tedious. However, this process will include routine technical training for technicians, monitoring of diagnostic accuracy by external QC procedures, outreach training, reference laboratory support, and regular proficiency testing to ensure comparable results among laboratories. An external QA plan is important to assure valid diagnostic results and accurate reporting. Harmonization of PCR procedures more recently has taken on higher priority given the increasing importance of molecular diagnostics in the ICEMR network and elsewhere in infectious diseases epidemiology, clinical care and research. Access to reference laboratories to assure accurate and available QA/QC in both malaria microscopy and PCR should be readily available, as a matter of public health policy within malaria-endemic regions.

Although this article focused on different diagnostic platforms and protocol standardization, being able to harmonize data across platforms is essential, in the case of the ICEMRs, enabling comparable case definitions across the ICEMRs for which diagnostics lead to interventions, whether for disease or surveillance.

## Figures and Tables

**Table 1:** Currently available tools to detect malaria parasites

Platform	Target	Description and potential use
Microscopy	Whole parasite	Detects asexual and sexual blood stage parasites of all species under microscope
		Reliable readings require skilled microscopists
RDT	Antigen	Detects malaria antigen by immunochromatographic assay with monoclonal antibodies to target parasite antigen
		Detect parasite antigens (HRP, aldolase, or pLDH) circulating in the blood
PCR (conventional and real time)	DNA	Amplifies target parasite DNA. Depending on the target gene, genus- and species-level diagnoses are available. The result of conventional PCR is qualitative whereas qPCR is quantitative
RT-PCR	RNA	Detects mRNA expressed at specific life cycle of the parasite. The test can be used to measure the transmissibility of the infection by quantifying the presence of mosquito-infective sexual stages
NASBA	RNA	Amplifies target RNA in a single step isothermal condition
LAMP	DNA	Detecting infection by a turbidity meter after amplifying parasite DNA; DNA extraction methods are key
Microarrays	DNA	Use extract parasite DNA on a hybridization platform to quantify parasitemia by fluorescence-based detection
Mass spectrometry	Heme	Detects infection based on identification of heme by laser desorption mass spectrometry
Flow cytometry	Hemozoin	Detects infection based on hemozoin concentration
Automated blood cell counter	Hemozoin	Detects infection based on malarial pigment in activated monocytes
Serological tests	Malaria parasite specific antibody	Detects and measures antibodies against malaria parasites as an indicator of recent and/or past exposure to parasites

HRP = histidine-rich protein; LAMP = loop-mediated isothermal amplification; NASBA = nucleic acid sequence–based amplification; pLDH = *Plasmodium* lactate dehydrogenase; RDT = rapid diagnostic test; qPCR = real-time quantitative PCR; RT-PCR = reverse transcriptase PCR.

**Table 2:** Detail of diagnostic tools and the usage at each ICEMR

		Amazonia	East Africa	India	Latin America	Malawi	Southeast Asia	Southern Africa	Southern Asia	Southwest Pacific	West Africa
Microscopic diagnosis	Use as a diagnostic tool	Yes	Yes	Yes	Yes	Yes	Yes	Yes	Yes	Yes	Yes
	Used as point-of-care diagnosis	Yes	Yes	Yes	Yes	Yes	Yes	No	Yes	No	Yes
	Quantification metric	WHO guidelines	WHO guidelines	WHO guidelines	WHO guidelines	WHO guidelines	WHO guidelines; percent parasitemia	WHO guidelines	WHO guidelines; percent parasitemia	WHO guidelines	WHO guidelines
	Differentiates asexual and gametocyte stages	Asexual (all together); sexual (all together)	Asexual (all together)	Asexual (all together); sexual (all together)	Asexual (all together); sexual (all together)	Asexual (all together); sexual (all together)	Asexual (all together); sexual (all together)	Asexual (all together); sexual (all together)	Asexual stages (all together); merozoites, trophozoites, schizonts (separately); sexual stages (all together); male and female gametocytes (separately)	Asexual (all together); sexual (all together); male and female gametocytes (separately)	Asexual (all together); trophozoites (separately); sexual (all together)
	QA/QC practices	Double reading; independent reading of 10% of slides in reference laboratory	Double reading; triple for discrepancies	Double reading; triple for discrepancies; diagnostic PCR	Double reading	Double reading; triple for discrepancies	Double reading	Double reading; triple for discrepancies	Double reading; triple for discrepancies; diagnostic PCR	Double reading; triple for discrepancies; independent reading of 10% of slides in reference laboratory; diagnostic PCR	Double reading; triple for discrepancies
RDT diagnosis	Use as a diagnostic tool	No	Yes	Yes	Yes	Yes	Yes	Yes	Yes	Yes	Yes
	Used as point-of-care diagnosis	Not applicable	Yes, cross-sectional studies	Yes, cross-sectional studies	No	Yes, based on availability	Yes, based on availability	Yes	Yes	Yes	Yes
	RDT(s)	Not applicable	Paracheck-*Pf*™	FalciVax	SD Bioline	SD One-Step	BinaxNOW^®^, Alere	SD Bioline (Zambia); ICT (Zimbabwe)	FalciVax	CareStart™, Apacor	Paracheck; First Response; SD Bioline
	Detection type	Not applicable	HRP2: *Plasmodium falciparum*	HRP2: *P. falciparum*; pLDH: *Plasmodium vivax*	HRP2: *P. falciparum*; pLDH: *P. vivax*	pLDH: *P. falciparum* and Pan	HRP2: *P. falciparum*; pLDH: Pan	HRP2: *P. falciparum*	HRP2: *P. falciparum*; pLDH: *P. vivax*	HRP2: *P. falciparum*; pLDH: Pan	HRP2: *P. falciparum*
	Species specificity	Not applicable	*P. falciparum*	*P. falciparum* and *P. vivax*	*P. falciparum* and *P. vivax*	*P. falciparum*, *P. vivax*, *Plasmodium malariae*, and *Plasmodium ovale*	*P. falciparum*, *P. vivax*, *P. malariae*, and *P. ovale*	*P. falciparum*	*P. falciparum* and *P. vivax*	*P. falciparum*, *P. vivax*, *P. malariae*, and *P. ovale*	*P. falciparum*
	Determining factor for selection of RDT(s)	Not applicable	Government recommendation; availability	Bivalent; efficacy results; availability	Government recommendation	Government recommendation; availability	Bivalent; availability	Government recommendation	Bivalent; availability	Government recommendation; sensitivity to *P. vivax*	Government recommendation; availability
	Performed RDT efficacy study	Not applicable	No	Yes	Yes	No	No	No	Yes	No	Yes
	Genotyping of parasite population for mutations that may impede accurate RDT diagnosis	Not applicable	No	No	No	HRP2 deletions	No	HRP2 deletions	No	No	HRP2 deletions
Molecular diagnosis	Use as a diagnostic tool	No	Yes	Yes	Yes	Yes	Yes	Yes	Yes	Yes	Yes
	Used as point-of-care diagnosis	No	No	No	No	No	No	No	Yes (LAMP)	No	Yes
	Molecular diagnostic(s) used	Conventional PCR; qPCR; RT-PCR	Conventional PCR	Conventional PCR	qPCR, RT-PCR, LAMP	qPCR, RT-PCR	Conventional PCR	Conventional PCR; qPCR; RT-PCR	Conventional PCR; LAMP	Conventional PCR; qPCR; RT-PCR	Conventional PCR; qPCR
	Morphological stage-specific molecular diagnostic(s)	Asexual and sexual	Asexual	Asexual	Asexual and sexual	Asexual and sexual	Asexual	Asexual and sexual	Asexual	Asexual and sexual	Asexual
	Target	18S rRNA	18S rRNA	18S rRNA	18S rRNA	pfLDH	18S rRNA	PfCytb, Pfs25	18S rRNA	18S rRNA, pfs25 and pvs25	18S rRNA, Pfr364
	Assays performed on-site or in centralized location	Onsite in Iquitos (Loreto) and Lima (Lima Province)	Onsite in Kampala	Onsite in Raurkela (Odisha), Chennai (Tamil Nadu), Nadiad (Gujarat); QA/QC in centralized location in Dwarka, New Delhi	Real-time PCR in centralized laboratory, LAMP in field	Centralized location in Blantyre	Centralized location in host countries	Onsite in Macha and Ndola (Zambia) and Harare (Zimbabwe)	Onsite in India	Onsite in Papua New Guinea; offsite at the Walter and Eliza Hall Institute and the Swiss Tropical and Public Health Institute	Centralized location in Mali (Senegal)

HRP2 = histidine-rich protein 2; LAMP = loop-mediated isothermal amplification; pLDH = *Plasmodium* lactate dehydrogenase; QA = quality assurance; QC = quality control; qPCR = quantitative polymerase chain reaction; RDT = rapid diagnostic test; RT-PCR = reverse transcriptase polymerase chain reaction; WHO = World Health Organization.

**Table 3:** DNA extraction methods for various starting materials

Starting material	Method	Reference
Whole blood	GTC preparation with subsequent phenol:chloroform extraction	44
	Rapid boiling method	45
	PURE method (followed by LAMP)	46
	Commercial kits (*Qiagen DNeasy Blood and Tissue Kit*, *QIA Amp Kit*, bioMérieux easyMag^®^, Abbott m2000)	47,48
Dried blood spot	*Chelex boiling*	49–52
	Commercial kits (*Qiagen DNeasy Blood and Tissue Kit*, *QIA Amp Kit*, bioMérieux easyMag^®^)	53,54
	Tris-EDTA protocol	55
	PURE method (followed by LAMP)	46
Thick blood smear	Qiagen mini kit with minor modification	56
RDT cassette	Boiling nitrocellulose component of the RDT strip in molecular grade water	57
Urine/saliva	Qiagen DNeasy Kit	58

EDTA = ethylenediaminetetraacetic acid; GTC = guanidine isothiocyanate; LAMP = loop-mediated isothermal amplification; RDT = rapid diagnostic test.

Method reported to be in use by ICEMRs are given in italics.
